# Oral Insulin Delivery Using Poly (Styrene Co-Maleic Acid) Micelles in a Diabetic Mouse Model

**DOI:** 10.3390/pharmaceutics12111026

**Published:** 2020-10-27

**Authors:** Fatemah Bahman, Sebastien Taurin, Diab Altayeb, Safa Taha, Moiz Bakhiet, Khaled Greish

**Affiliations:** Department of Molecular Medicine, Princess Al-Jawhara Centre for Molecular Medicine, School of Medicine and Medical Sciences, Arabian Gulf University, Manama 328, Kingdom of Bahrain; sebastient@agu.edu.bh (S.T.); diabad@agu.edu.bh (D.A.); safat@agu.edu.bh (S.T.); moiz@agu.edu.bh (M.B.)

**Keywords:** diabetes, insulin, nanomedicine, SMA, oral delivery

## Abstract

The oral delivery of insulin is a convenient and safe physiological route of administration for management of diabetes mellitus. In this study, we developed a poly-(styrene-co-maleic acid) (SMA) micellar system for oral insulin delivery to overcome the rapid degradation of insulin in the stomach, improve its absorption in the intestine, and provide a physiologically-relevant method of insulin to reach portal circulation. The insulin was encapsulated into SMA micelles in a pH-dependent process. The charge and size of the nanoparticles were determined by dynamic light scattering. The insulin loading of the nanoparticles was measured by HPLC. The transport of the SMA-insulin through biological membranes was assessed in vitro using Caco-2 cells, ex vivo rat intestinal section, and in vivo in a streptozotocin-induced diabetes mouse model. SMA-insulin micelles were negatively charged and had a mean diameter of 179.7 nm. SMA-insulin efficiently stimulated glucose uptake in HepG-2 hepatic cells and was transported across the Caco-2 epithelial cells in vitro by 46% and ex vivo across intestinal epithelium by 22%. The animal studies demonstrated that orally-administered SMA-insulin can produce a hypoglycemic effect up to 3 h after administration of one dose. Overall, our results indicate that SMA micelles are capable of the oral delivery of bioactive compounds like insulin and can be effective tools in the management of diabetes.

## 1. Introduction

Diabetes mellitus (DM) is the most common metabolic disease and the seventh leading cause of death globally, according to the World Health Organization [1]. Either insufficient insulin production or the inability to use the insulin produced promotes its development. DM is concerned with multiple organ-related complications, such as the kidneys in the urinary system and the cardiovascular system, as well as causing damage to the retina of the eyes and nerves throughout the body [2]. DM patient cases have swelled from 171 to 382 million from 2000 to 2013 and are projected to increase up to 642 million worldwide by 2040 [3,4]. There are two major forms of the disease, T1DM and T2DM, both characterized by hyperglycemia. Other less frequent forms include gestational DM and genetic alterations of β-cells [5]. T1DM accounts for 5–10%, while T2DM cases represent up to 90% of DM cases [6]. As a standard method, subcutaneous insulin injection is commonly used in patients with DM to normalize the blood glucose level (BGL) once or multiple times per day. Subcutaneous injections, however, are often associated with poor compliance where numerous regular injections can lead to hypertrophy, infections, and lipid deposits at the injection sites as well as skin allergy and production of exogenous insulin antibodies [7]. Therefore, oral administration of insulin could improve disease management, enhance patient compliance, and decrease the long-term complications of DM [8]. The main obstacles to oral insulin delivery are the proteolytic enzyme activities and harsh pH conditions in the stomach, and the mucus layer in the small intestine, leading to unpredictable absorption and poor bioavailability [9]. Recently, several alternative insulin delivery routes involving nanotechnologies have been exploited to overcome the limitations of the standard delivery [10,11].

The use of nanomaterials of different types, for instance, hydrogels, liposomes, and micelles, have enabled oral insulin delivery to overcome the barriers of the gastrointestinal tract (GIT) [12,13,14,15]. Various strategies were explored for improving oral delivery of insulin, for example, the use of enhancers for permeation, mucoadhesion, protease inhibitors, and polymeric drug delivery carriers [14,16,17]. Natural polymers, like chitosan that complexed with oleic acid, and labrasol (PEG 8 caprylic/capric glycerides), a surfactant, was used for the delivery of insulin and lowered BGL significantly with *p* < 0.05 after 3 h and up to 12 h in diabetic rats compared to subcutaneous injection [18]. Several derivatives, such as carboxylated, carboxymethyl, dimethylethyl, trimethylated, and thiolated chitosan, have been used to formulate nanoparticles (NP) with improved oral bioavailability and decreasing blood glucose level (BGL) [19,20,21]. Additionally, Sarmento et al., formulated NP from a dilute alginate solution with insulin that reduced the relative basal glucose concentration in the blood by more than 40% in diabetic rats receiving the equivalent of 50 and 100 IU/kg insulin; the sustainability of the hypoglycemic impact was recorded for up to 18 h [22]. Moreover, Chalasani et al., developed an insulin-VitB12 dextran conjugate NP (20 IU/kg) which significantly lower of BLG for at least 24 h [23]. Synthetic polymers, like poly (lactic-co-glycolic acid) (PLGA) [24] and polylactic acid (PLA) [25], have been used in oral insulin delivery systems with promising results. Furthermore, coupled PLGA to cell-penetrating peptides (CPP) were established to enable insulin absorption [26]. Another colloidal particle known as solid lipid nanoparticles (SLN) used as protection shield for insulin against gastric enzymatic activity and enhances GIT absorption due to its mucoadhesive effect. Thus, cetyl palmitate-based SLNs were layered with poloxamer 407 to protect the insulin NP against aggregation in simulated gastric fluid (SGF) and reduced the BGL in diabetic male Wistar rats by 20% over 24 h [27]. In this study, we used a synthetic polymeric micelle (PM) for the oral delivery of insulin because the NP of biodegradable polymers have been developed to be delivered orally for protein and peptide drugs. Moreover, these nanoparticles have the advantage to cross the GIT and to be taken by the M-cells of Peyer’s patches within the intestinal tract [28]. When critical micelle concentration (CMC) is achieved, PM are formed spontaneously in aqueous solution by the assembly of the amphiphilic copolymer blocks. The diameters of these PM range from a few to hundreds of nm [29]. PM have improved durability and gradual dissociation, which allow the controlled release of loaded drugs. Due to the reactivity of the anhydride groups, several maleic copolymers have been proposed as macromolecular carriers. The shell-forming polymers are designed to extend blood circulation periods, escape the reticuloendothelial system, and promote drug targeting. In comparison to liposomes, PM are prepared using simple and low-cost techniques and allow a controlled delivery [30]. PM, such as poly-(styrene-co-maleic acid) (SMA) copolymers, consist of a hydrophobic styrene moiety and hydrophilic maleic acid segment [31]. SMA is a synthetic co-polymer block composed of styrene and maleic anhydride with an average molecular weight of 1600. SMA has demonstrated a potential for oral delivery and is proven to traverse intestinal barriers with appropriate therapeutic outcomes [32]. The SMA micellar system was used previously for the oral delivery of anticancer agents [32,33]. The SMA system is especially well-suited for different applications, due to the low cost of the polymer, simplicity of the preparation process, and the loading can be controlled (up to 40% *w/w*) [33].

In this study we developed SMA polymeric micelles encapsulating insulin (SMA-insulin) and assessed its biological activity in vitro and in vivo in a diabetes mouse model. The SMA-insulin micelle loading, charge, and size were characterized. We demonstrated that the encapsulation did not impair the activity of insulin in vitro and decreased the BGL in diabetic mice.

## 2. Materials and Methods

### 2.1. Materials

Poly-(styrene-co-maleic anhydride), cumene terminated (average MW ~1600), *N*-(3-dimethylaminopropyl)-*N*-ethylcarbodiimide hydrochloride (EDAC), Hank’s balanced salt solution, advanced Dulbecco’s Modified Eagle’s Medium (DMEM), Roswell Park Memorial Institute (RPMI) 1640 medium, fetal bovine serum (FBS), bovine serum albumin (BSA), and TrypLE Express were purchased from ThermoFisher Scientific (Dubai, United Arab Emirates). Human recombinant insulin (91077C) was purchased from Merck (Hertfordshire, UK). L-glutamine, penicillin/streptomycin, glucose uptake fluorometric assay Kit, 4-(2-hydroxyethyl)-1-piperazineethanesulfonic acid (HEPES), and pepsin were purchased from Merck (Hertfordshire, UK). All consumables such as petri dishes, plastic tubes (15 mL and 50 mL), cell culture flasks (25 cm^2^ and 75 cm^2^), transwell plates (CLS3460), and simulated gastric fluids (cat no. 1651) were purchased from Merck (Hertfordshire, UK).

### 2.2. Methods

#### 2.2.1. Synthesis of SMA-Insulin

Briefly, the SMA was prepared in a four-steps process as described previously [34]. SMA is synthesized by the copolymerization of styrene and maleic anhydride monomers. First, the hydrophobic poly (styrene-co-maleic anhydride) was hydrolyzed in alkaline conditions (0.5 M NaOH) under a constant stirring at 70 °C overnight. Second, insulin was encapsulated into the SMA micelles. Human recombinant insulin (150 mg) was initially dissolved in 1 mL distilled water (DW) at pH 2 and *N*-(3-dimethylaminopropyl)-*N*-ethylcarbodiimide hydrochloride (EDAC) (450 mg) in DW. EDAC solution, a catalyst that reacts with the carboxyl bonds of SMA, and insulin solution were added simultaneously to the SMA solution (pH 5) under constant stirring. The pH was continuously maintained at 5 using 0.1 N HCl and stirred until stabilization of the pH. Third, the solution was gradually adjusted to pH 11 with 0.1 N NaOH under constant stirring and until the pH has stabilized. As insulin was encapsulated, the pH was then adjusted to 7.4 with 0.1 N HCl. Fourth, the SMA-insulin micelles were purified and concentrated using a Labscale ultrafiltration system with a 10 kDa Pellicon XL filter (Merck Millipore, Hertfordshire, UK). The concentrated micelle solution was lyophilized to obtain a SMA-insulin powder stored at 4 °C. The lyophilization process starts when the temperature is close to (−60 °C) and the pressure is 5 mTorr. The lyophilizer run for four days until complete lyophilization.

#### 2.2.2. Characterization of SMA-Insulin

##### Loading of the SMA Micelles

The loading of SMA micelles was determined by dispersing the SMA-insulin micelles at 1 mg/mL in DW. Various insulin loadings were achieved by using different ratios of SMA over insulin. Insulin loading was established by comparison to a standard curve of the free insulin established by reverse phase high performance liquid chromatography (RP-HPLC) and measured at 214 nm. The amount of insulin loaded was obtained from the following equation:

y = 0.0052x − 0.0026; (y = Absorbance; x = Concentration of insulin (μg/mL))(1)

The concentration of insulin in each sample was established, and the % of insulin expressed as the total amount of insulin detected/weight of the nanoparticles × 100.

###### Size, Polydispersity Index (PDI), and Zeta Potential Determination of SMA Micelles

Lyophilized SMA-insulin powder weighted with minimum dose (4 mg/mL) was dissolved in sodium bicarbonate (0.1 M, pH 7.4) which will protect the micelle from precipitation and enable to determine the nanomicellar size and polydispersity index (PDI), precisely. The charge was assessed by dissolving (4 mg/mL) of the SMA-insulin in distilled water (DW) at pH 2. All measurements for size distribution and zeta potential were performed using the Malvern ZEN3600 Zetasizer nano series (Malvern Instruments Inc., Westborough, MA, USA) based on dynamic light scattering (DLS). Measurements were obtained from three independent experiments performed in triplicate.

#### 2.2.3. In Vitro Drug Release Profile at Physiological pH and in Simulated Gastric Fluid (SGF)

SMA-insulin solution (12 mg in 1.5 mL) was placed in a dialysis bag with a molecular weight cut off of 12 kDa and dialyzed against distilled water physiological pH (pH 7.4), or SGF with pepsin, (pH 1.2) (15 mL). At specific time points, samples (2 mL) outside the dialysis bag were removed, and the absorbance was measured at 214  nm by HPLC [35]. The release rate was expressed as the ratio of absorbance between the solution outside the bag at defined time points and the absorbance within the bag at t = 0.

The percentage of release of insulin from the SMA-insulin micelles was determined as follows:

% Release = (Amount outside the bag (at defined time)/(Amount inside the bag at t = 0 min) × 100.

#### 2.2.4. Cell Culture

Caco-2 and HepG-2 cells were obtained from (ATCC, Manassas, VA, USA). Caco-2 cells are human epithelial colorectal adenocarcinoma cells used as a model of the intestinal barrier [32]. HepG-2 cells are a human hepatocellular liver carcinoma cell line used to measure glucose uptake [36]. Both cell lines were maintained in advanced DMEM supplemented with 10% FBS, 2 mM L-glutamine, 100 units/mL penicillin, 100 µg/mL of streptomycin, and 2.2 g/L of sodium bicarbonate in an incubator set at 37 °C with a humidified atmosphere containing 5% carbon dioxide (CO2). For all procedures, cells were harvested using TrypLE Express.

#### 2.2.5. In Vitro Model for Evaluating the Intestinal Transport of SMA-Insulin Micelles

Caco-2 cells were seeded (80,000 cells/cm^2^) onto polycarbonate transwell filters with a 0.4 μm mean pore size and diameter of 12 mm (Merck Millipore, Hertfordshire, UK). Caco-2 cells were maintained for 21 days with the media being changed every other day. Transepithelial electrical resistance (TEER) was measured in each well with an epithelial voltohmmeter to ascertain the formation and the integrity of the monolayer. Monolayers with a TEER > 350 Ω/cm^2^ were used for the evaluation of SMA-insulin transport. Cell monolayers were washed with Hank’s balanced salt solution (HBSS) supplemented with HEPES (1 mM, pH 7.4). SMA-insulin solution (100 μM) was added on the top of the differentiated Caco-2 cell monolayer. After incubating for 3 h, TEER was measured, and media from the lower compartment was collected. Transport of the SMA-insulin was quantified by measuring the absorbance of insulin in the media of the lower compartment at 214 nm.

#### 2.2.6. In Vitro Model for Evaluating the Glucose Uptake of SMA-Insulin Micelles

Glucose uptake was assessed using the glucose uptake fluorometric assay kit, and according to the manufacturer protocol. HepG-2 cells were seeded at a density of 60,000 cells/cm^2^ onto a 25 cm^2^ flask and grown until differentiation. Then, HepG-2 cells were starved in serum-free DMEM overnight and then equilibrated for 40 min in Krebs-Ringer-Phosphate-Hepes buffer. Subsequently, cells were stimulated with an equivalent concentration SMA-insulin and free insulin (1 µM) for 1 h. Then, 2-deoxyglucose (2-DG) (10 mM) was added for an additional 20 min. Cells were washed three times with PBS and lysed with extraction buffer provided with the kit, then frozen at −80 °C for 10 min and heated at 85 °C for 40 min. After cooling on ice, neutralization buffer was added, and lysates centrifuged. The remaining lysate was then diluted with assay buffer (MAK084B). Lastly, the fluorometric end-product generation was set up in two steps according to the kit manufacturer’s instructions and then detected at (λex = 535/λem = 587 nm). Subsequently, the differentiated HepG-2 cells monolayer glucose uptake was evaluated by fluorescence intensity using confocal (ZEN 2009-Confocal microscopy LSM 710 (Zeiss, Oberkochen, Germany).

#### 2.2.7. Ex Vivo Model for Evaluating the Transport of SMA-Insulin Micelles

To evaluate the transport of SMA-insulin micelles through the intestinal epithelium, a rat everted gut sac ex vivo model was used [37]. The intestine was obtained from male Sprague-Dawley rats, 4–6 months of age. The animal protocol was approved by the Arabian Gulf University animal ethics committee (E020-PI-11/18, 9 January 2017). The entire small intestine was excised, irrigated several times with HBSS, and immediately placed at 37 °C in HBSS with carbogen supply. The intestine was everted over a glass rod and divided into 1 cm sections of duodenum, jejunum, and ileum. These sections were ligated at one end, filled with HBSS (1 mL), and then ligated at the other end. These everted sacs were immersed into HBSS (10 mL) containing solubilized SMA-insulin (100 µM) and maintained with carbogen supply for the duration of the experiments. The transport of SMA-insulin from mucosal to serosal surfaces was determined after 3 h of incubation by measuring absorbance at 450 nm using enzyme-linked immunosorbent assay (ELISA) kits (ab200011; Abcam, Cambridge, UK) and following the manufacturer recommendations.

#### 2.2.8. In Vivo Model to Evaluate the Effect of SMA-Insulin Micelles on Healthy and Diabetic C57BL/6 Mice

All the animal procedures were approved by the Arabian Gulf University animal ethics committee. Male C57BL/6 mice (5–6 weeks old) were obtained from the Animal House at the Arabian Gulf University. Mice were housed in pathogen-free conditions at 21–24 °C on a scheduled 12 h light/dark cycle, with food and water ad libitum. Mice were randomly divided into six groups (G1-G4; *n* = 10 and G5, G6; *n* = 6) and treated with either free insulin or an equivalent dose of SMA-insulin. BGL was measured using a glucometer, as described in Table 1. Furthermore, we assessed the effect of SMA-insulin on the BGL in diabetic mice. All mice were intraperitoneally (IP) injected with streptozotocin (STZ) (60 mg/kg) freshly prepared in citrate buffer, pH 4.5 [38]. When BGL rose above 300 mg/dL, mice were randomly divided into three groups of three animals, as described in Table 2. Two different doses of SMA-insulin equivalent to 36 and 72 mg/kg were given orally and BGL assessed.

### 2.3. Statistical Analysis

Data are presented either as the mean ± SD or the mean ± SEM. Experiments were performed in triplicate or more as indicated for each data set in the text or figure legends. For statistical analysis, t-tests were employed to compare two groups. One-way analysis of variance (ANOVA) test were used to assess the differences between multiple groups in the animal studies. Statistical analyses were performed using GraphPad Prism 8 software (GraphPad Software, CA, USA). *p*-value < 0.05 was considered statistically significant.

## 3. Results

### 3.1. Characterization of SMA-Insulin

The synthesized SMA-insulin micelles had a recovery of 78% and loading of 18% of insulin as determined by the weight ratio of insulin over SMA. The mean diameter of SMA micelles, measured by DLS was 179 ± 38.9 nm. The PDI of SMA micelles was 0.27. The zeta potential of the SMA-insulin was −0.979 mV, as presented in (Table 3).

### 3.2. In Vitro Drug Release Profile at Physiological pH and in Simulated Gastric Fluid (SGF)

**Figure 1:**
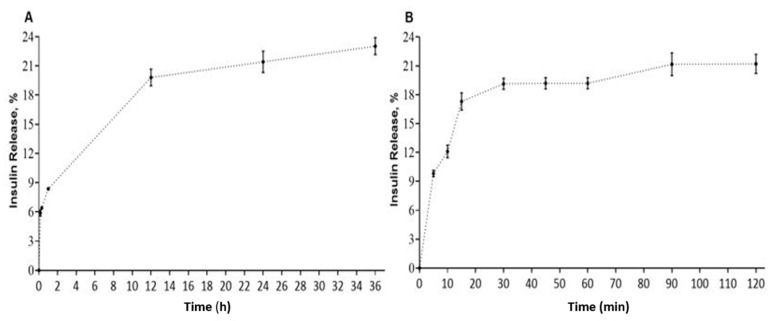
SMA-insulin micelles release rate. (**A**) In vitro release of insulin from SMA-insulin was assessed over 36 h, at pH 7.4, using a dialysis method. (**B**) In vitro release of insulin from SMA-insulin in SGF at pH 1.2 over 120 min using a dialysis method. Absorbance was measured at 214 nm. Data expressed as means ± SEM (*n* = 3).

At physiological pH, the release of insulin from SMA-insulin micelles increased to 19.8% during the first 12 h and plateaued at 23% after 36 h (Figure 1A). In simulated gastric fluid, an initial burst release of 9.8% was measured, which then increased steadily to 21% after 2 h, a period corresponding to the gastric transit time (Figure 1B).

### 3.3. In Vitro Model for Evaluating the Transport of SMA-Insulin Micelles

**Figure 2:**
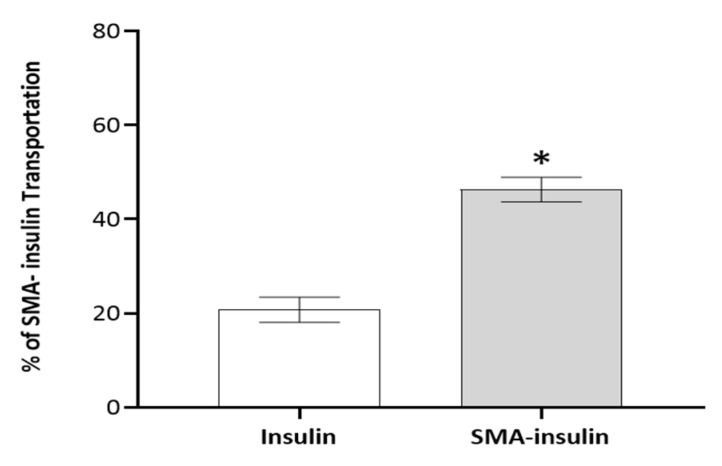
SMA-insulin micelles transportation across in vitro model of the intestinal epithelium. The differentiated Caco-2 cell monolayers were incubated with insulin and SMA-insulin solutions (100 μM) to the apical surface for 3 h. Data are expressed as means ± SEM (*n* = 4). % of total transport of insulin was analyzed using a *t*-test. * indicates statistical difference from the insulin group, *p* < 0.05.

The ability and efficacy of SMA-insulin micelles to traverse the intestinal epithelium cell lining were measured in vitro using a differentiated Caco-2 cell monolayer. Caco-2 cells were grown for 21 days on a transwell to form a cell monolayer that phenotypically resembles the enterocytes lining the intestinal epithelium [39]. The encapsulation of insulin facilitates the transport of insulin from the apical to basal side of the differentiated Caco-2 cell monolayer. Up to 46% of insulin of the original SMA-insulin dose applied apically was detected in the lower compartment of the transwell, while only 20% of the free insulin dose was measured. Insulin content in the lower compartment of the transwell was measured by HPLC using a UV detector at 214 nm (Figure 2).

### 3.4. In Vitro Model for Evaluating the Glucose Uptake of SMA-Insulin Micelles

**Figure 3:**
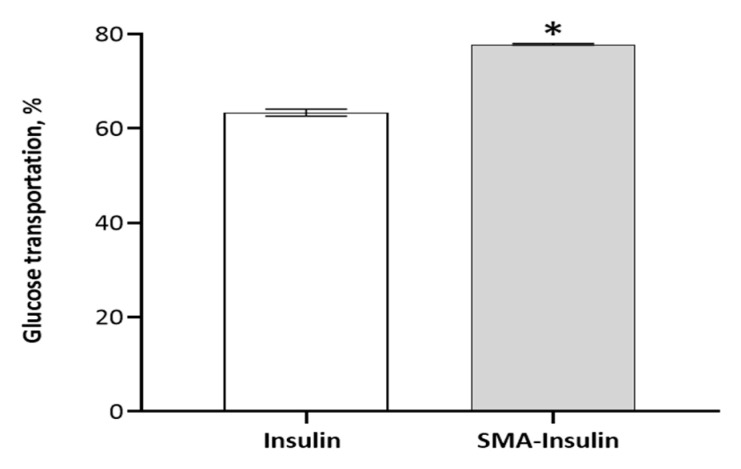
Glucose uptake of insulin and SMA-insulin micelles. HepG-2 cells were starved in DMEM without serum overnight, followed by 40 min of incubation in KRPH buffer. Then, HepG-2 cells were treated with SMA-insulin and free insulin (100 µM), simultaneously to the addition of 2-DG (10 mM) to the media. The 2-DG uptake was assessed over 1 h. The accumulation of fluorometric end-product generation of 2-DG was measured using λex = 535/λem = 587 nm. Data are expressed as the mean ± SEM (*n* = 4). * indicates a statistical difference from the insulin group, *p* < 0.05.

**Figure 4:**
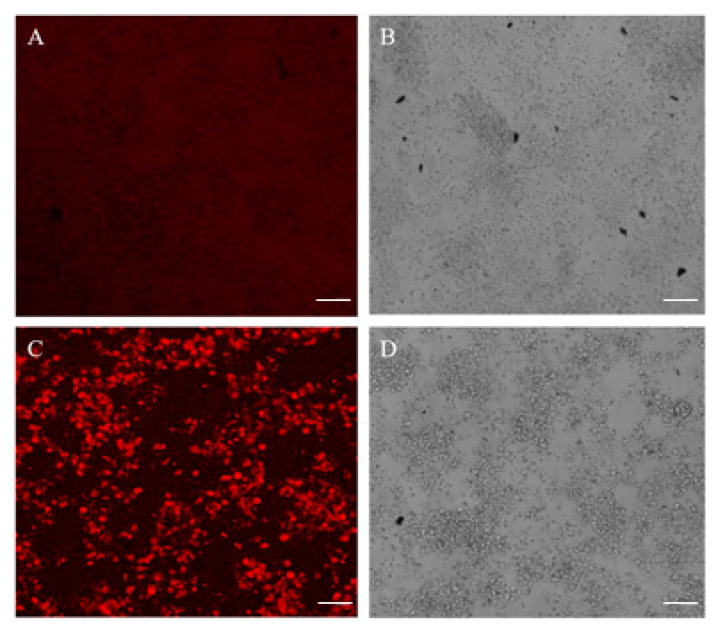
Glucose uptake induced by SMA-insulin micelles. Confocal images of fluorescent 2-DG uptake in HepG-2 cells untreated (**A**,**B**) and SMA-insulin treated (**C**,**D**) for 3 h. HepG-2 cells were seeded in a six-well plate for two days. HepG-2 cells were starved before the addition of 10 mM of fluorescent 2-DG. These pictures were done in triplicate; representative images are shown. Scale bar 200 µm.

The effect of SMA-insulin micelles on the absorption of glucose was evaluated in vitro using differentiated HepG-2 cells. The cellular uptake of a non-metabolizable glucose analog, 2-DG, was measured in the presence of free insulin and SMA-insulin (100 μM). The SMA-insulin treatment increased the uptake of 2-DG by 15% when compared to free insulin over 1 h (Figure 3). The long-term effect of SMA-insulin (100 μM) on glucose transport was further demonstrated by confocal microscopy using fluorescent 2-DG. After 3 h incubation with SMA-insulin at room temperature of 37 °C, the HepG-2 cells accumulated a large amount of fluorescent 2-DG as shown by the high fluorescence intensity (Figure 4C), while control cells absent of insulin failed to accumulate 2-DG (Figure 4A).

### 3.5. Ex Vivo Model for Evaluating the Transport of SMA-Insulin Micelles

**Figure 5:**
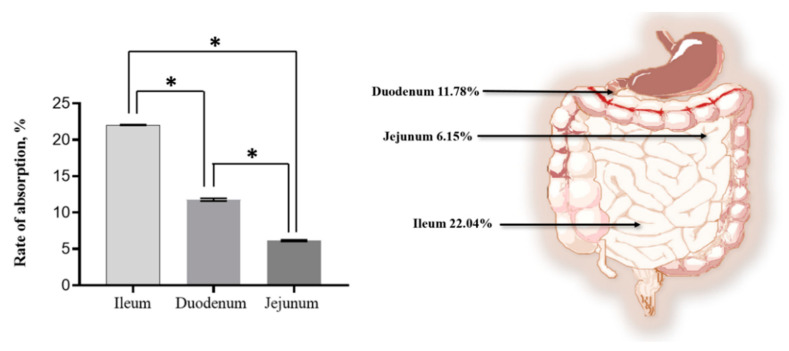
SMA-insulin micelle transportation across an intestinal rat ex vivo model. Transport of SMA-insulin through the rat duodenum, jejunum and ileum sections following a 4 h incubation. Quantification of insulin was performed using an ELISA kit and following the manufacturer instructions. Absorbance was measured at 450 nm. Data are expressed as mean ± SEM (*n* = 6). The percentage of total SMA-insulin was analyzed using a one-way analysis of variance (ANOVA), * *p* < 0.05 denotes statistical significance.

The SMA-insulin transportation across the intestine was assessed using an everted rat small intestine sac model. The transportation of SMA-insulin was measured from the mucosal side to the serosa side of the everted rat small intestine sacs and quantified by a human insulin ELISA kit. The ileum sections had a significantly higher transport efficacy of insulin of 22.04%, while the duodenum and jejunum sections demonstrated a lower insulin transport capacity of 11.78 and 6.15%, respectively (Figure 5).

### 3.6. In Vivo Evaluation of Oral SMA-Insulin Micelles Effect on BGL of Healthy C57BL/6 Mice

**Figure 6:**
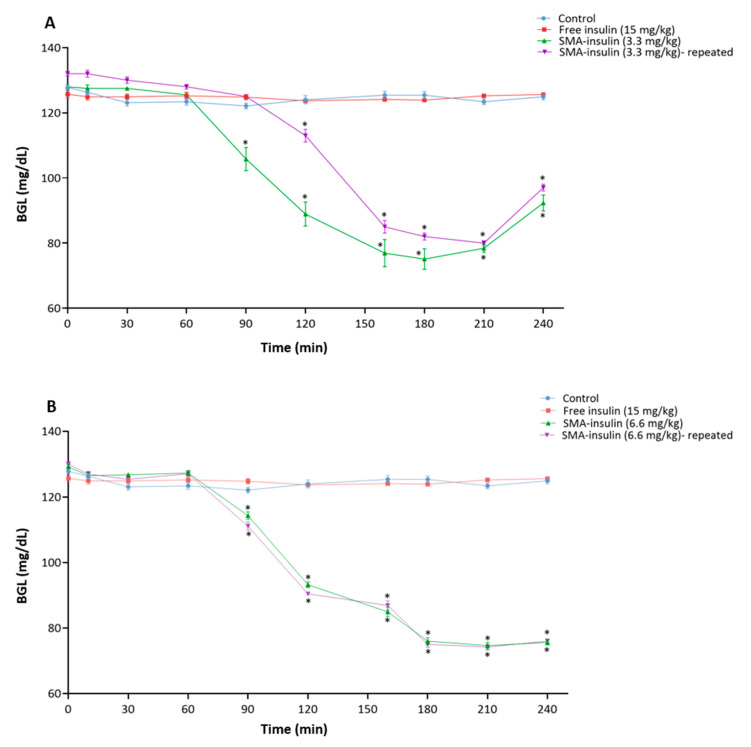
Blood glucose profiles of healthy mice following oral administration of SMA-insulin micelles and insulin. Mice (*n* = 10 per group) were fasted overnight before oral gavage with water, insulin (15 mg/kg) and either (**A**) SMA-insulin (3.3 mg/kg) single or repeated dosing (24, 48 and 72 h) or (**B**) SMA-insulin (6.6 mg/kg) single or repeated dosing (24, 48 and 72 h). The BGL was measured for 4 h using a glucometer. Data are represented as mean ± SD (*n* = 10). The mean of BGL was analyzed using a one-way ANOVA. * *p* < 0.05 denotes statistical significance.

The effect of the oral administration of free insulin and SMA-insulin on BGL was initially assessed in healthy mice starved overnight (Figure 6). The free insulin (15 mg/kg) given orally did not demonstrate any alteration of the BGL in healthy animals (Figure 6A). However, SMA-insulin with 3.3 mg/kg had a significant effect on lowering BGL from 128 ± 2 to 75 ± 9.8 mg/dL up to 3.5 h then started to increase after 4 h of administration. A double dose 6.6 mg/kg also significantly decreased the BGL from 129.2 ± 2 to 74.6 ± 3.2 mg/dL and the lowering were sustained for more than 4 h (Figure 6B). Moreover, the effect of SMA-insulin was dose-dependent, as the doubling of the dose prolongs the hypoglycemic effect (Figure 6B). Furthermore, the repeated dosing after 24, 48, and 72 h with the same orally given doses of SMA-insulin 3.3 and 6.6 mg/kg exhibited a similar effect on lowering BGL (Figure 6A,B).

### 3.7. In Vivo Evaluation of Subcutaneous Injection SMA-Insulin Micelles Effect on BGL of Healthy C57BL/6 Mice

**Figure 7:**
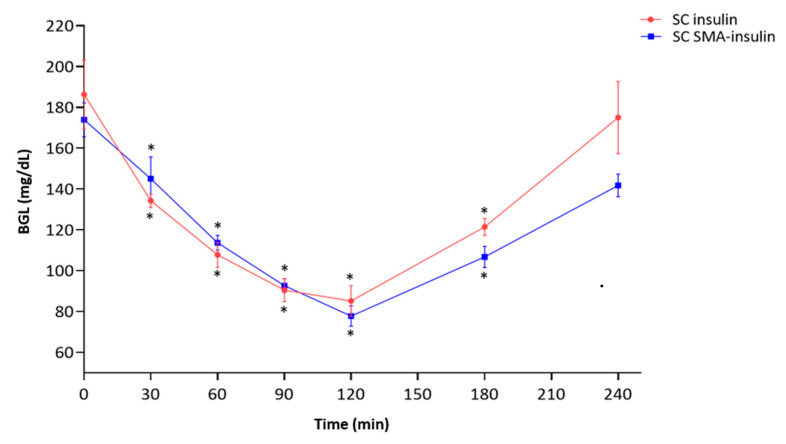
BGL profiles following subcutaneous administration of insulin and SMA-insulin. Animals fasted overnight before insulin or SMA-insulin (2 mg/kg) subcutaneous injection. BGL was measured for 4 h at regular intervals using a glucometer. Data are stated as mean ± SD (*n* = 6/group). The mean of BGL was analyzed using a one-way ANOVA * *p* < 0.05 denotes statistical significance.

SMA-insulin (2 mg/kg) and free insulin (2 mg/kg) were injected subcutaneously (SC) in non-diabetic mice (*n* = 6 per group) to assess their effect on the BGL. The SC free insulin triggered a hypoglycemic effect in healthy mice, and decreased BGL from 186.3 ± 16.8 to 85.1 ± 9.2 mg/dL. The SMA-insulin administered subcutaneously had almost the same hypoglycemic effect, which reduced BGL from 174 ± 8.4 to 76.5 ± 4.5 mg/dL (Figure 7). Interestingly, by comparing BGL of SC injection of free insulin and SMA-insulin at each specific time point showed that both have a similar profile and were not statistically significant (*p*-value > 0.05).

### 3.8. In Vivo Evaluation of Oral SMA-Insulin Micelles Effect on BGL of Diabetic C57BL/6 Mice

**Figure 8:**
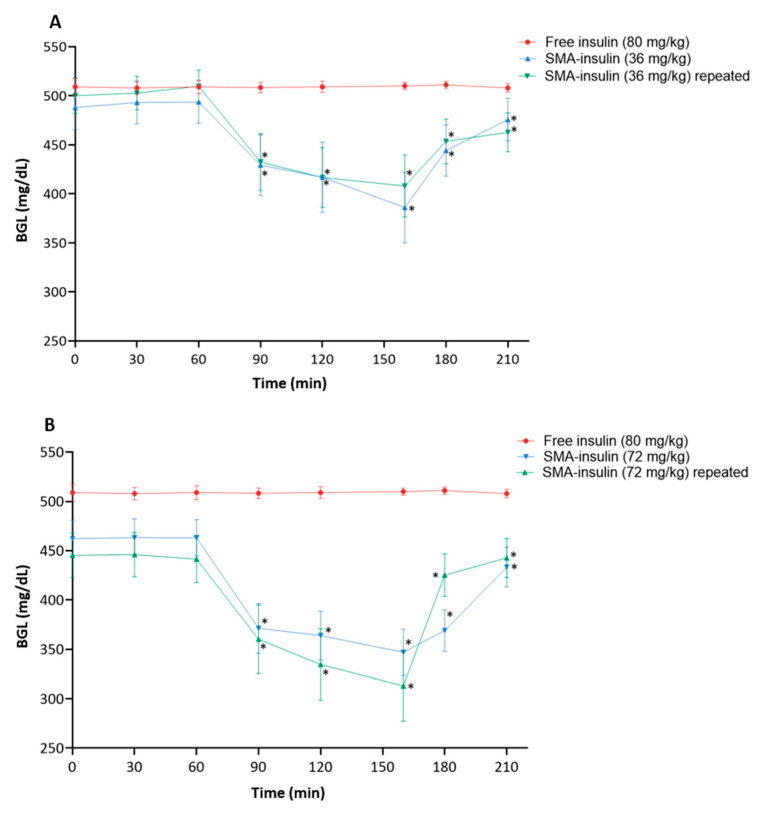
BGL profiles following the oral administration of SMA-insulin (36 and 72 mg/kg) or insulin (80 mg/kg) in STZ induced diabetic mice. Diabetic mice were fasted overnight before the oral administration of either insulin (80 mg/kg) or (**A**) SMA-insulin single and repeated doses (36 mg/kg), or (**B**) SMA-insulin single and repeated doses (72 mg/kg). The BGL was measured for 4 h at different time intervals using a glucometer. Data are presented as mean ± SD (*n* = 6). The mean of BGL was analyzed using a one-way ANOVA. * *p* < 0.05 denotes statistical significance when compared to the free insulin oral administration.

Diabetic mice were treated with either SMA-insulin or free insulin at different doses by oral gavage. Oral administration of the free insulin (80 mg/kg) did not reduce the BGL. In diabetic mice, the administration of SMA-insulin (36 mg/kg) and (72 mg/kg) lowered the BGL from 488 ± 55 to 386 ± 88 mg/dL and from 445 ± 55 to 313 ± 87 mg/dL, respectively (Figure 8). When compared with the oral administration free insulin, both doses of SMA-insulin (36 mg/kg and 72 mg/kg) reduced significantly the BGL (*p* < 0.05). The repeated doses of SMA-insulin approved the reduction of the BGL (Figure 8A,B). The doubling of the dose from 36 to 72 mg/kg tend to indicate statistically significant (*p* < 0.05) dose-dependent effect on BGL as well (Figure 8B).

## 4. Discussion

The parenteral route of insulin delivery is the mainstay treatment for DM type I and few patients with type II who require lifelong repeated injections. Although various non-parenteral routes of administration of insulin have been studied, the oral delivery of insulin remains the most convenient route among patients. Oral delivery of insulin is very beneficial compared with subcutaneous injections as it reduces patient discomfort, improves patient quality of life, and could maintain blood glucose levels. Still, the oral route of insulin faces daunting challenges as a result of the degradation and digestion by proteolytic enzymes in the stomach and the low permeability of the intestinal epithelium to insulin.

The encapsulation of insulin into nanoparticles is providing new opportunities for the control of insulin delivery. Many nanocarrier systems, such as liposomes and polymeric nanoparticles, have been developed to improve bioavailability and prolong the stability of their payload, yet there is no clinically-available oral insulin nanomedicine for DM management [40,41].

For effective oral insulin delivery, these nanocarriers should be stable in the gastrointestinal environment, able to penetrate the intestinal mucosa, be transported across the intestinal epithelium to reach the systemic circulation, and release the bioactive insulin. In this study the SMA micellar system was evaluated as nanocarriers for oral insulin delivery. SMA is characterized as water soluble with amphiphilic features, biocompatible and biodegradable micelle carriers of hydrophobic drugs that form noncovalent binding to the plasma albumin facilitating its circulation and prolonging in the plasma. Ideal physiochemical characteristics of the SMA micellar system, specifically its amphiphilic nature, has allowed the encapsulation of a wide range of hydrophobic drugs, such as pirarubicin [42], doxorubicin [34], zinc protoporphyrin [43], Caveolin-1 [33], and epirubicin [32]. Therefore, the encapsulation of insulin into SMA is a pH-dependent process, as demonstrated in a previous publication [34]. The encapsulation of insulin may be facilitated by the alteration of its conformation at low pH, promoting the nucleation of the protein through the association of the hydrophobic domains [44], and eventually the interaction with the styrene entities of the SMA. Our in vitro and in vivo results tend to indicate that the insulin is likely to be physically encapsulated inside the core of the micelle out of reach of proteases.

Size is an important parameter for the enhancement of cellular uptake and delivery of the encapsulated drug. Thus, the common DLS method is used for nanoparticle size characterization. It uses time-dependent light scattering fluctuations as a result of the Brownian motion of the particles to determine the size of the nanoparticle [45]. The average hydrodynamic diameter of SMA-insulin nanoparticles was 179.7 ± 38.9 nm. Other studies have been conducted and support that the SMA-insulin micelles size were able to be taken up by intestinal cells, such as Banerjee et al., who demonstrated that the smaller sized nanoparticles between 50 nm to 200 nm were taken up efficiently by intestinal cells, and more efficiently than larger-sized nanoparticles [46]. Additionally, enterocytes have been shown to effectively uptake nanoparticles between 100 and 200 nm [47]. He et al. reported that nanoparticles less than and equal to 300 nm in size are suitable for oral drug delivery because M-cells and enterocytes favorably internalized them [48]. Moreover, the PDI value for SMA-insulin was 0.27, demonstrating the homogeneity of the micelle preparation. The average zeta potential observed for SMA-insulin was slightly negative, at –0.979 mV, attributed primarily to the carboxyl-terminated maleic acid, and within the acceptable range for electrostatic stability [49]. The charge of the nanoparticle is also a factor to consider for the intestinal cell uptake [50]. Several studies demonstrated that charged particles (positive or negative) are taken up better than uncharged [51]. The negatively-charged nanoparticles, such as SMA-insulin, are likely to be taken by caveolae-mediated endocytosis with some exceptions, while neutral nanoparticles show no preferred route for endocytosis [52]. The transcytosis of nanoparticles is mediated by the caveolae and is likely to be influenced by the charge of the nanoparticles [53]. Several important factors affect the drug loading capacity of micelles, such as the nature of the total copolymer molecular weight, core forming block, core block length, nature and concentration of the drug, as well as the nature and block length of the outer micelle shell (corona) [54]. In our case, the styrene part of SMA rearranges to form a hydrophobic interaction with hydrophobic components in insulin by stable pH adjustments, yielding stable nano-formation with high drug loading up to 18% of insulin loading.

To understand the effect of pH on the release mechanism of insulin, we performed the release rate experiment under varying pH conditions (pH 1.2 and 7.4). The release rate of insulin from SMA micelles at pH 7.4 was 19.8% during the first 12 h and increased up to 23% after 36 h. However, when SMA-insulin was suspended in SGF (pH 1.2) with pepsin, an initial burst release of 9.8% was observed, which increased steadily to 21% after 2 h. The human gastric emptying time is typically 80.5 ± 22.1 min for liquids and 143.6 ± 54.6 min for solids [55]. The initial burst release observed at acidic pH may be due to a conformation change due to the alteration of the charges of insulin and SMA, which promotes the weakly-bound insulin to escape from the micelle. At pH 7.4, the micellar formulation is more stable and should favor the slow release of insulin in the systemic circulation. The self-assembled SMA polymeric micelles involve dynamic interactions that condition their equilibrium and influence their kinetics over time.

The SMA-insulin transport through the epithelium lining in the intestine was evaluated using an established in vitro Caco-2 cell model. The in vitro model allows the evaluation of the transport of nanoparticles through paracellular, diffusion, endocytosis, or transcytosis [15]. In the present study, the permeability of the “differentiated Caco-2 enterocyte mimic” to free insulin is limited and may involve simple diffusion with a combination of non-specific internalization at the membrane [56]. The transcellular transport process of SMA-insulin from the apical side to the basolateral side is likely to involve either single or multiple pathways, including micropinocytosis, micropinocytosis, caveolin-mediated endocytosis, and clathrin-mediated endocytosis. A previous publication using SMA loaded with epirubicin identified clathrin-mediated endocytosis and micropinocytosis in the transport of SMA-epirubicin micelles across the differentiated Caco-2 cell monolayer [32].

The everted rat small intestine sac model provides evidence of the SMA-insulin absorption [57]. The small intestine consists of three regions duodenum, jejunum, and ileum, where the pH gradually increases from 6 in the duodenum to about 7.4 in the terminal ileum. These three regions differ mainly by the shape of the villi, the proportion of goblet cells, and the presence of Peyer’s patches. The Peyer’s patches are absent in the duodenum and jejunum but present in the ileum. Peyer’s patches are also called gut-associated lymphoid tissue (GALT) and are composed of the aggregations of lymphoid tissue and contain specialized microfold cells (M-cells) [58]. The primary function of M-cells is to sample antigens from the lumen of the small intestine and deliver them via transcytosis to antigen-presenting cells and lymphocytes [58]. A previous study showed that SMA micelles carrying a fluorescent marker were co-localized with M-cells [32]. Insulin nanoparticles can interact with M-cells via both specific and non-specific receptor-mediated mechanisms [59]. The transportation of insulin nanoparticles through the M-cells will overcome pre-systemic hepatic metabolism and enhance drug bioavailability. In this study, the results showed that the ileum section has significantly higher transportation of SMA-insulin of 21.8% while, the duodenum and jejunum sections demonstrated about 12% and 6.6%, respectively. Our results indicate that the uptake of the SMA-insulin may involve both enterocytes and M-cells, as a portion of the intestine responsible for the highest transport efficiency accounts for the most substantial proportion of M-cells. After nanocarrier uptake, two significant hypotheses can be considered [60]. The first hypothesis is that free or encapsulated insulin transported to the capillary vessels through the villi and then carried to the portal circulation reaches the liver. Then, insulin is eventually processed in the first-pass metabolism by the hepatic enzymes before it reaches systemic circulation similarly to the physiologic insulin. The second hypothesis involved the transport of SMA-insulin by the mesenteric lymph nodes located in the villi and next to the blood vessels, then carried to the systemic circulation by the thoracic duct. At this point, insulin nanocarriers might act similarly to subcutaneously injected insulin [61].

Further, to confirm the ability of SMA-insulin to protect the insulin from proteolytic degradation in vivo studies were carried out. The oral administration of SMA-insulin (3.3 and 6.6 mg/kg) in healthy mice shows a significant hypoglycemic effect. In a DM type I model, STZ induced diabetic mice were given a single oral dose of free insulin (80 mg/kg) or SMA-insulin at 36 or 72 mg/kg. While free insulin failed to modify the BGL level, the oral administration of SMA-insulin at a dose of 36 or 72 mg/kg decreased the BGL to a maximum of ~21 and 30%, respectively, after 160 min in STZ induced diabetic mice. Additionally, this effect was not sufficient to induce a major hypoglycemic effect and the return to a normal BGL in diabetic mice; these results demonstrated the potential of SMA for the oral delivery of insulin. Previously published works have demonstrated the feasibility of the oral administration of insulin. Chitosan-pentasodium tripolyphosphate nanoparticles were shown to maintain BGL for 11 h in diabetic rats with a dose of 100 IU/kg [31], chitosan-poly (γ-glutamic acid) decreased and maintained BGL up to 10 h in diabetic rats with a dose 30 IU/kg [62], insulin-S.O (Ins-S.O) complex reduced BGL to 23.85% for up to 12 h post-oral administration and the effect was prolonged for 24 h [63], dichloromethane, ethyl acetate containing 2% polymer (*w*/*v*) added to the insulin-phospholipid (Ins-SPC) complex nanoparticles reduced the fasting BGL to 57.4% within the first 8 h and this continued for 12 h [64], and VB12-dextran nanoparticle system reduced BGL up to 24 h. It is undeniable that our system is less efficient and will require further development, though the encapsulation process of insulin into SMA is simple, inexpensive, modulizable, and biocompatible, Furthermore, this effect was not sufficient to induce a major hypoglycemic effect and the return to a normal BGL in diabetic mice; these results demonstrated the potential of SMA for the oral delivery of insulin.

## 5. Conclusions

The present study explored the oral delivery of insulin using SMA as a nanocarrier. The encapsulation of insulin into the SMA did not affect its biological activity, promoted the transport of glucose in vitro, and protected it for the harsh environment in the stomach. Moreover, the SMA-insulin decreased the BGL of STZ-induced diabetic mice. The SMA-insulin micelle will require further optimization to improve its absorption in the intestine and transfer to the systemic circulation. Additionally, the structure of SMA is amendable to targeted moieties to facilitate its adhesion to the mucus and transport through the intestinal barrier.

## Figures and Tables

**Table 1:** Insulin and SMA-insulin treatment groups for non-diabetic mice.

Group No.	Treatment/Dose	Administration Route
1-Healthy mice	DW	Oral gavage
2-Healthy mice	Free Insulin 15 mg/kg	Oral gavage
3-Healthy mice	SMA-insulin 3.3 mg/kg	Oral gavage
4-Healthy mice	SMA-insulin 6.6 mg/kg	Oral gavage
5-Healthy mice	Free insulin 2 mg/kg	SC injections
6-Healthy mice	SMA-insulin 2 mg/kg	SC injections

**Table 2:** Insulin and SMA-insulin treatment groups for diabetic mice.

Group No.	Treatment/Dose	Administration Route
1-Diabetic mice	DW	Oral gavage
2-Diabetic mice	SMA-insulin 36 mg/kg	Oral gavage
3-Diabetic mice	SMA-insulin 72 mg/kg	Oral gavage

**Table 3:** SMA–insulin preparation characteristics.

	Recovery	Loading (*wt/wt*)	Size (nm)	PDI	Zeta Potential (mV)
SMA-insulin	78%	18%	179.7 ± 38.9	0.27	−0.979 ± 0.0346

Data are shown as the mean ± standard deviation (SD). Measurements were performed in triplicate and represent the average of three different preparations. PDI = polydispersity index.
